# Aortoenteric Fistula: An Uncommon but Life-Threatening Complication of Pledget Use in Hiatal Hernia Repair

**DOI:** 10.7759/cureus.81432

**Published:** 2025-03-29

**Authors:** Jeet H Patel, Parag Brahmbhatt, Kalpit Devani, Jena Velji-Ibrahim

**Affiliations:** 1 Medicine, Medical University of Silesia, Katowice, POL; 2 Gastroenterology, Eagle Physicians, Greensboro, USA; 3 Gastroenterology, Prisma Health-Upstate/University of South Carolina School of Medicine, Greenville, USA; 4 Resident, Prisma Health-Upstate/University of South Carolina School of Medicine, Greenville, USA

**Keywords:** aortoenteric fistula, laparoscopic fundoplication, laparoscopic hiatal hernia repair, laparoscopic nissen fundoplication, pledget

## Abstract

The standard treatment for hiatal hernia repair and gastroesophageal reflux disease (GERD) is laparoscopic Nissen fundoplication, in which a Teflon pledget (TP) is used to buttress the hiatal hernia. We present an extremely rare case in which a 74-year-old female developed a postoperative aortoenteric fistula due to erosion of a TP used during paraesophageal hernia repair. A computed tomography (CT) angiogram and esophagogastroduodenoscopy (EGD) confirmed the diagnosis. Subsequently, a thoracic endovascular aortic repair (TEVAR) was performed to treat the aortoenteric fistula, and the TP was removed using a raptor grasping device and scissors during a repeat EGD at a tertiary care center. In this article, we discuss different causes of TP erosion and possible countermeasures that may help prevent such complications.

## Introduction

Laparoscopic Nissen fundoplication has become a standard treatment for hiatal hernia repair and gastroesophageal reflux disease (GERD). A Teflon pledget (TP) is used in this procedure as a buttress under sutures to prevent tearing of the tissue, particularly in areas containing blood vessels, and to distribute tension evenly. According to previous studies, approximately 90% of patients undergoing laparoscopic fundoplication have had positive outcomes, and postoperative TP-related complications are rare. Among those rare cases, common late postoperative complications include gas-bloat syndrome (up to 85%), dysphagia (10%-50%), diarrhea (18%-33%), and recurrent heartburn (10%-62%) [1-6]. We present an extremely rare case in which a patient developed postoperative acute anemia due to an aortoenteric fistula caused by the TP used during paraesophageal hernia repair. A review of the current literature has not revealed any similar cases related to laparoscopic Nissen fundoplication.

## Case presentation

A 74-year-old female was referred to a general surgeon for a paraesophageal hernia repair due to a history of refractory/recurrent episodes of LA grade C esophagitis with previous significant nonsteroidal anti-inflammatory drug (NSAID) use and GERD-related symptoms. Proton pump inhibitors (PPIs) were unsuccessful in relieving her of heartburn and regurgitation symptoms. She had undergone multiple endoscopies in the past, which indicated moderate to large hiatal hernia/paraesophageal hernia. 

On January 24, 2024, she underwent robotic reduction of paraesophageal hiatal hernia with type II mediastinal dissection, primary repair of hiatal hernia, anterior and posterior gastropexy, Toupet fundoplication, and mesh reinforcement with surgical TPs. 

On March 13, 2024, she presented due to a sudden episode of dizziness and nausea in the emergency department. Her vitals at that time were measured as blood pressure (BP): 101 mmHg/85 mmHg (reference range systolic: <120 mmHg, diastolic: <80 mmHg), pulse (P): 118 beats per minute (reference range 60-100 beats per minute), and respiratory rate (RR): 22 breaths per minute (reference range 12-20 breaths per minute). The lab results were normal except for her hemoglobin levels, which were 11.9 g/dL (reference range: 12.1-15.1 g/dL). She was subsequently discharged from care and sent home with conservative management. On March 21, 2024, she returned complaining of severe back pain and nausea. She presented with BP: 109 mmHg/67 mmHg (reference range systolic: <120 mmHg, diastolic: <80 mmHg), P: 72 beats per minute (reference range 60-100 beats per minute), and RR: 24 breaths per minute (reference range 12-20 breaths per minute). On further inquiry, she also complained of black/dark stools but was on iron supplementation. The lab results at the time were normal except for her hemoglobin levels, which were 5.2 g/dL (reference range: 12.1-15.1 g/dL), and her lactic acid levels were greater than 9 mmol/L (reference range <2 mmol/L). Subsequent computed tomography (CT) angiogram revealed findings suspicious for contained gastroesophageal perforation versus developing/early outer gastroesophageal fistula with abundant blood product in the lumen of the stomach. After a surgical consultation, esophagogastroduodenoscopy (EGD) was performed, and one large cratered esophageal ulcer with no active bleeding was discovered, 35 cm from the incisors. The ulcer seemed suspicious for a visible vessel or aorto-esophageal fistula; however, it was decided that advancing the scope further should be avoided as it could disrupt the clot and initiate bleeding.

Furthermore, the patient was also seen by a vascular surgeon whose interpretation of the CT angiogram indicated a concerning aortoenteric fistula, which may have been the cause of her prior bleeding episodes. Subsequently, the patient underwent an urgent thoracic endovascular aortic repair (TEVAR) for possible aortoenteric fistula as well as celiac artery stenting. Thereafter, she was given total parenteral nutrition (TPN) and had a gastrostomy tube (G-tube) placement on March 27, 2024, to avoid further complications. Nevertheless, she developed a peripherally inserted central catheter (PICC) line-associated deep vein thrombosis (DVT) requiring Eliquis therapy and was additionally placed on lifelong antibiotics due to the aortic stenting. 

**Figure 1:**
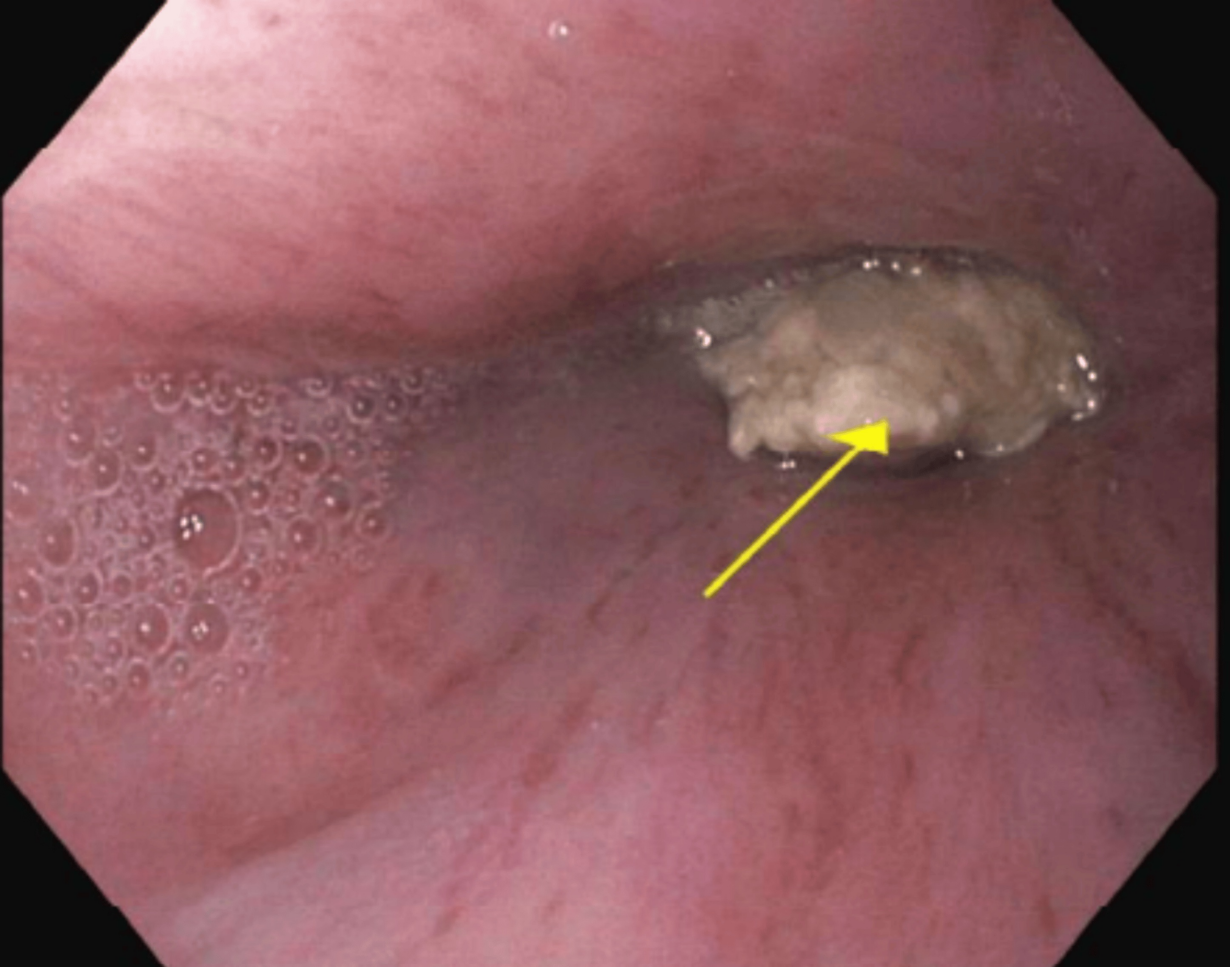
The arrow indicates the superficial border of the Teflon pledget discovered during repeat esophagogastroduodenoscopy at a tertiary care center.

**Figure 2:**
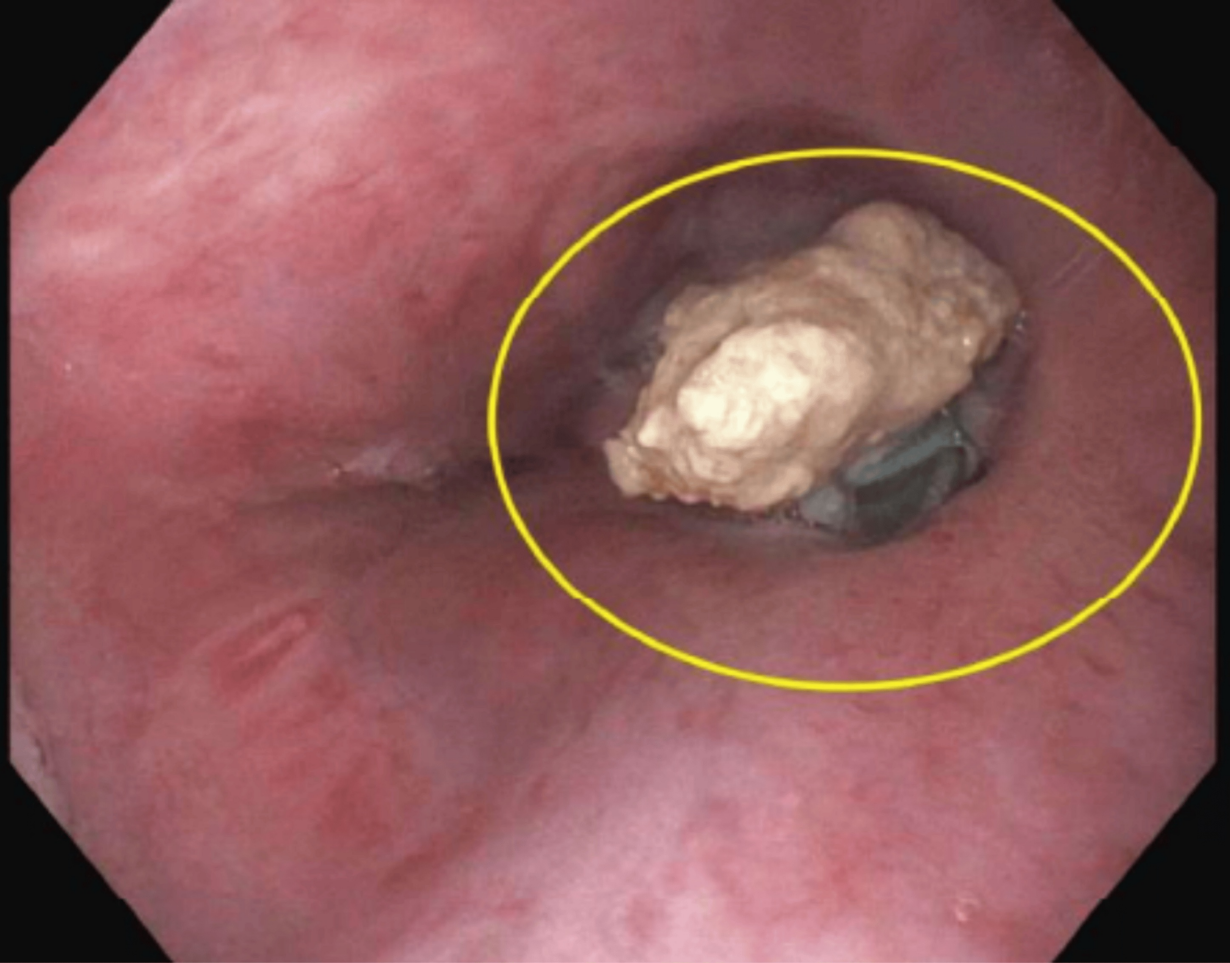
The white structure in this figure is the eroded Teflon pledget discovered during the repeat esophagogastroduodenoscopy at a tertiary care center.

In August of 2024, she underwent a repeat EGD at a tertiary care center and was found to have a large suture TP (Figure 1, 2) implanted at 37 cm, which was removed with a raptor grasping device and scissors. The patient has been stable since, indicating that the pledget erosion was the cause of the aortoenteric complication, which led to her acute anemia episodes.

## Discussion

The patient presented with acute anemia-related symptoms. An EGD and CT angiogram revealed an incidence of aortoenteric fistula. A repeat EGD confirmed the cause of the aortoenteric fistula to be the erosion of a TP used during the paraesophageal hernia repair procedure. TP-related complications are rare. Thus, finding relevant articles discussing this issue poses a challenge. A PubMed search, using the parameters “Teflon pledget and fistula” and “Teflon pledget and erosion” for a timeline between 1980 and 2024 revealed only two relevant articles. The first article involved a case report in which purulent drainage was noted from the formation of a cardiocutaneous fistula due to a TP that had been used six years earlier on the heart suture line for left ventricular aneurysm repair [7]. The second relevant article involved a case report where a patient presented with postoperative dysphagia because of an esophagogastric fistula, which was caused by the erosion of a TP four weeks after a laparoscopic fundoplication procedure [8]. No relevant literature regarding patients presenting with acute anemia due to TP use was found, further demonstrating how rare this case is.

As discussed previously, fistula formation due to TP is an extremely rare phenomenon, and the cause of TP erosion remains unclear. One possible explanation could be ischemic necrosis caused by over-compact suturing. In one case study, a Teflon membrane placed anterior to the uterus to prevent adhesions post-myomectomy eroded into the bladder four years later. In that case, they speculated that ischemia was due to the use of a compact suture to retain the patch, which eventually led to fistula formation and local sepsis [9].

Another possible cause of TP erosion could be infection resulting from the use of TP. A case report from 1999 presented a patient who developed a cardiocutaneous fistula extending through the left hemidiaphragm and draining at the abdominal wall six years after a ventricular aneurysmectomy. The cause of TP erosion, which led to the cardiocutaneous fistula, was theorized to be a delayed infection from the suture line involving TP [7].

Lastly, another possible explanation for this phenomenon could be an inflammatory reaction to the TP. A case report from 2000 presented a patient with postoperative dysphagia caused by an esophagogastric fistula resulting from the erosion of TP. In that case, the authors speculated that an inflammatory response to the TP may have contributed to the complication [8].

A possible solution to prevent erosion of pledgets could be to use absorbable pledgets, such as polyglycolic acid (PGA) pledgets. A review of the current literature revealed no studies in which absorbable pledgets were used for laparoscopic Nissen fundoplication. However, one study did explore the use of PGA pledgets to repair bronchus, lung fistula, and pleural defects in approximately 50 dogs. That evaluation suggested that PGA pledgets were a better alternative to conventional non-absorbable pledgets for pulmonary procedures [10].

## Conclusions

In sum, absent conclusive evidence, mere speculations regarding the causes of TP erosion are insufficient to establish the theoretical reasons discussed above. Furthermore, TP use to buttress hiatal hernia repair will likely remain part of the standard procedure, as most patients experience successful outcomes. However, despite the lack of literature and evidence surrounding the use of PGA pledgets, we believe that resorbable pledgets could be a potential future alternative to TP to reduce complications. We hope that this article draws attention from other surgeons who may face similar challenges.
